# Comparison of Emergency Room Visits and Rehospitalization for Bleeding Complications following Transurethral Procedures for the Treatment of Benign Prostatic Hyperplasia: A Population-Based Retrospective Cohort Study

**DOI:** 10.3390/jcm11195662

**Published:** 2022-09-26

**Authors:** Shih-Liang Chen, Chih-Kai Hsu, Chun-Hsiang Wang, Che-Jui Yang, Ting-Jui Chang, Yu-Hsuan Chuang, Yuan-Tsung Tseng

**Affiliations:** 1 Department of Urology, Tainan Municipal Hospital (Managed by Show Chwan Medical Care Corporation), Tainan City 701, Taiwan; 2 Department of Hepatogastroenterology, Tainan Municipal Hospital (Managed by Show Chwan Medical Care Corporation), Tainan City 701, Taiwan; 3 Department of Optometry, Chung Hwa Medical University, Tainan City 717, Taiwan; 4 Department of Urology, Chang Bing Show Chwan Memorial Hospital, Changhua 505, Taiwan; 5 Department of Medical Research, Tainan Municipal Hospital (Managed by Show Chwan Medical Care Corporation), Tainan City 701, Taiwan

**Keywords:** benign prostatic obstruction, transurethral resection of the prostate, laser surgery of the prostate, clot retention

## Abstract

Background: The postoperative bleeding complications associated with laser surgery of the prostate and transurethral resection of the prostate (TURP) were compared. Methods: We used the Taiwan National Health Insurance Research Database to conduct an observational population-based cohort study. All eligible patients who received transurethral procedures between January 2015 and September 2018 were enrolled. Patients who received laser surgery or TURP were matched at a ratio of 1:1 by using propensity score matching, and the association of these procedures with bleeding events was evaluated. Results: A total of 3302 patients who underwent elective transurethral procedures were included. The multivariable Cox regression analysis revealed that diode laser enucleation of the prostate (DiLEP) resulted in significantly higher emergency room risks within 90 days after surgery due to clot retention than the Monopolar transurethral resection of the prostate (M-TURP) (Hazard Ratio: 1.52; 95% Confidence Interval [CI], 1.06–2.16, *p* = 0.022). Moreover, GreenLight photovaporization of the prostate (PVP) (0.61; 95% CI, 0.38–1.00 *p* = 0.050) and thulium laser vaporesection of the prostate (ThuVARP) (0.67; 95% CI, 0.47–0.95, *p* = 0.024) resulted in significantly fewer rehospitalization due to clot retention than did M-TURP. No significant increase in blood clots were observed in patients using comedications and those with different demographic characteristics and comorbidities. Conclusions: Among the investigated six transurethral procedures for Benign prostatic hyperplasia, PVP and ThuVARP were safer than M-TURP because bleeding events and clot retention were less likely to occur, even in patients receiving anticoagulant or antiplatelet therapy. However, DiLEP and holmium laser enucleation of the prostate (HoLEP) did not result in fewer bleeding events than M-TURP.

## 1. Introduction

Benign prostatic hyperplasia (BPH) is the most prevalent chronic disease among middle-aged and older men worldwide [1]. BPH surgery is covered by the Taiwan National Health Insurance (NHI) program. Monopolar transurethral resection of the prostate (M-TURP) is the standard treatment for BPH with small-sized prostate glands. However, M-TURP is associated with short-term postoperative bleeding-related complications such as hematuria and clot retention [2]. Bipolar TURP (B-TURP) is a safe and effective procedure with a significantly shorter operating time and efficacy similar to that of conventional M-TURP [3].

The introduction of TURP was followed by the advent of laser technology, which led to the development of some therapies with greater efficacy and fewer complications than TURP. GreenLight photovaporization of the prostate (PVP), thulium laser vaporesection of the prostate (ThuVARP), holmium laser enucleation of the prostate (HoLEP), and diode laser (980 nm) enucleation of the prostate (DiLEP) are more efficient laser techniques compared to TURP, with reported reproducible clinical results and few bleeding-related complications [4,5,6].

Because of the increasing prevalence of comorbidities and indications, the number of patients with BPH undergoing transurethral laser procedures increased. Transurethral laser procedures are associated with lower intraoperative blood loss and fewer discharge days [7,8,9]. However, no definite conclusions were drawn because of insufficient evidence. Moreover, few studies compared severe bleeding tendencies associated with all transurethral laser procedures and TURP in the same baseline population. This study evaluated the postoperative bleeding complications among common laser techniques and TURP for BPH.

## 2. Materials and Methods

### 2.1. Data Sources

The National Health Insurance Research Database (NHIRD) is a population-level dataset derived from the claims data of more than 99% of people in Taiwan enrolled in the NHI program [10]. The research database includes data on patients’ medical history, medication use, surgical intervention history, personal data, and diagnosis identified on the basis of the International Classification of Diseases, Ninth and Ten Revision, Clinical Modification (ICD-9-CM and ICD-10-CM, respectively) diagnosis codes [11,12].

The study protocol was approved by the Research Ethics Committee of Show Chwan Memorial Hospital (IRB-No: 1091213), and the requirement for informed consent was waived because the NHIRD datasets contain no identifiable personal information.

### 2.2. Study Design

We conducted a nationwide cohort study by retrieving NHIRD data on hospitalized patients who underwent their first TURP or laser surgery between 2015 and 2018. Follow-up data before enrollment were used to evaluate comorbidities. The discharge date of patients after surgery was considered their index date.

In order to evaluate the postoperative status of patients, we performed a follow-up for 15, 30, 60, and 90 days after surgery and calculated the occurrence of emergency room (ER) visits and rehospitalizations during the postsurgical period. Furthermore, we determined the statistical differences between these rates according to patient characteristics and surgery type.

### 2.3. Surgery Types

The M-TURP uses a single active electrode at the surgery site with a non-conductive hypo-osmolar irrigation medium. The B-TURP incorporates monopolar technology and is performed in a normal saline environment, addressing the dilutional hyponatremia of conventional monopolar TURP [13,14].

Type of laser techniques includes PVP, ThuVARP, HoLEP, and DiLEP. Based on the different wavelength-dependent laser–prostatic tissue interactions, the main techniques are coagulation, vaporization, resection, and enucleation. Although each laser type is different in design, their principle is not distinctive [15,16].

**Figure 1:**
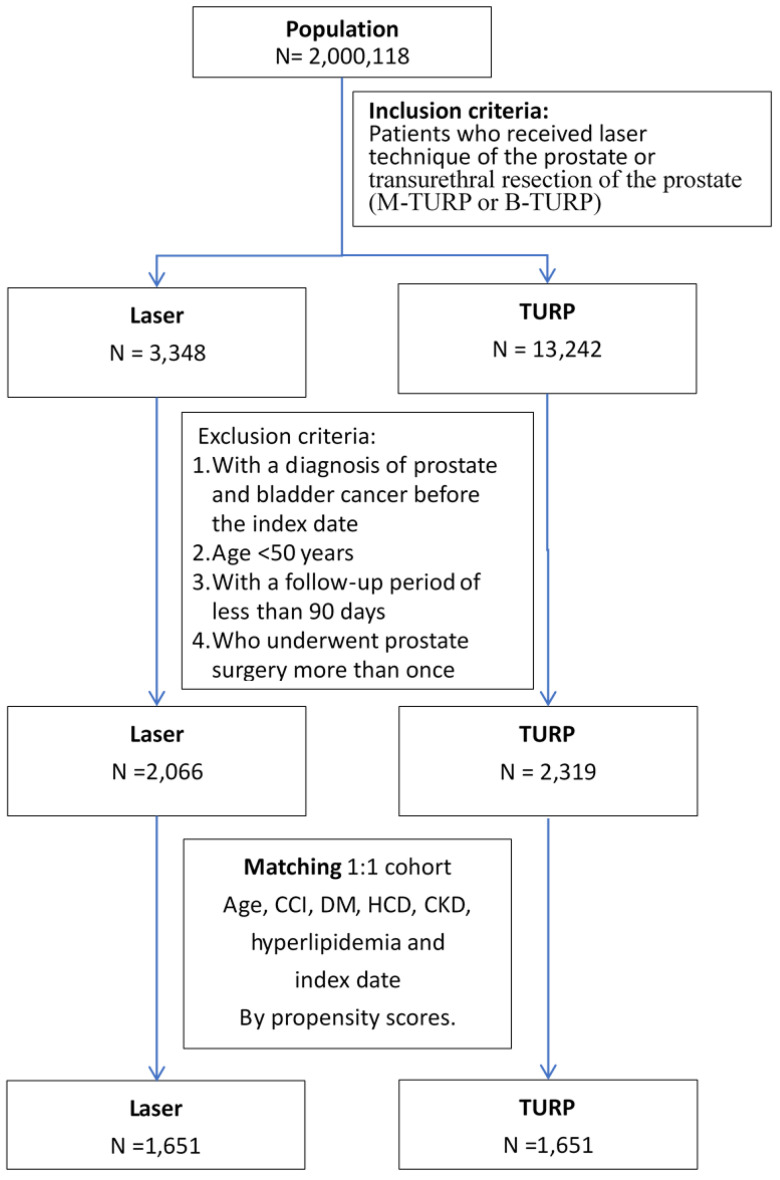
Flowchart of patient selection. Abbreviations: TURP: transurethral resection of the prostate; M-TURP: monopolar transurethral resection of the prostate; B-TURP: bipolar transurethral resection of the prostate; CCI: Charlson comorbidity index; HCD: hypertensive cardiovascular disease; DM: diabetes mellitus; CKD: chronic kidney disease.

Patients underwent either TURP (M-TURP or B-TURP) or transurethral laser procedures, namely PVP, ThuVARP, HoLEP, and DiLEP. The flowchart of patient selection and inclusion and exclusion criteria are provided in Figure 1.

### 2.4. Covariate Assessment

We identified the following covariates that are potential confounders: Charlson comorbidity index (CCI), age, and medication history. The medication history of the following drugs was assessed: statins, angiotensin-converting enzyme inhibitors (ACEIs), β-blockers, anticoagulant drugs (warfarin, rivaroxaban, apixaban, dabigatran etexilate, enoxaparin, tirofiban, heparin, and fondaparinux), antiplatelet drugs (aspirin, cilostazol, clopidogrel, dipyridamole, prasugrel, rivaroxaban, and ticlopidine), spironolactone, and 5-α-reductase inhibitors.

We also assessed the following comorbidities: diabetes mellitus (DM; ICD-9 CM code 250 and ICD-10 CM codes E10.0, E10.1, E10.9, E11.0, E11.1, and E11.9), hypertensive cardiovascular disease (HCD; ICD-9 CM codes 401–405 and ICD-10 CM codes I10–I15), chronic kidney disease (CKD; ICD-9 CM code 585 and ICD-10 CM code N18), hyperlipidemia (ICD-9 CM code 272 and ICD-10 CM code E78), heart failure (ICD-9 CM code 428 and ICD-10 CM code I50), bladder cancer (ICD-9 CM code 188 and ICD-10 CM code C67), and prostate cancer (ICD-9 CM code 185 and ICD-10 CM code C61).

### 2.5. Main Outcome Measurements

#### 2.5.1. Postoperative Bleeding Complications Leading to ER Visits

We evaluated the bleeding events leading to ER visits in patients with urine retention, acute urine retention, hematuria, using tranexamic acid, and who underwent diagnostic or treatment procedures, including bladder sonography, bladder instillation, bladder irrigation with a foley catheter, or bladder blood clot evacuation with a Toomey bladder evacuator.

#### 2.5.2. Postoperative Bleeding Complications Leading to Rehospitalization

We evaluated the bleeding events leading to rehospitalization in patients using tranexamic acid and who underwent diagnostic or treatment procedures, including bladder sonography or cystoscopy (the inspection of the bladder and urethra with cystoscopy and removal of clots with suction), bladder blood clot evacuation with a Toomey bladder evacuator, bladder instillation, and intermittent or continuous bladder irrigation with a foley catheter.

### 2.6. Statistical Methods

We performed between-group comparisons by using the paired *t*-test [17] for continuous variables and McNemar’s [18] test for categorical variables. Cox regression [19] analysis with covariates was used to estimate the relationship and differences in the risk of bleeding between the TURP and laser surgery groups. The hazard ratios (HRs) and 95% confidence intervals (CIs) for the outcomes were measured for all groups. The Kaplan–Meier method was used to estimate the outcomes of the study cohorts. The differences between the curves were examined using the log-rank test [20]. 

#### Propensity Score Matching

Propensity score matching (PSM) is a popular approach for estimating treatment effects by using observational data [21]. In order to reduce selection bias and the effects of confounders, we used robust PSM to create matched sets of patients who underwent TURP and those who underwent laser surgery at a ratio of 1:1 with full matching without replacement. Logistic regression was used for propensity score calculation [22]; the covariates used in the logistic regression model were age, DM, HCD, CKD, hyperlipidemia, CCI, and the index year at the start of the follow-up. The flowchart of surgery type and matching is presented in Figure 1.

Baseline characteristics were matched using PSM to reduce potential selection bias. PSM was performed using multivariate logistic regression, and matching was performed using the package of R Statistical Software “MatchIt” (version 4.4.0; R Core Team 2021, Vienna, Austria). Statistical analysis was performed using SPSS 21.0 (SPSS Inc., Chicago, IL, USA), and statistical significance was set at *p* < 0.05.

## 3. Results

### 3.1. Patient Characteristics

Patients with noncancerous BPH who underwent surgery between 2015 and 2018 were included in this study (Figure 1). We performed PSM and included 3302 patients; of them, 1651 underwent laser surgery, and 1651 underwent B-TURP or M-TURP. The mean age and CCI of patients were 70.9 ± 8.4 years and 3.2 ± 2.6. The demographic characteristics of the TURP group and laser surgery groups are listed in Table 1. Patients in both groups were men with similar age and comorbidity distributions. No differences in common event-related comorbidities and medication history were observed between the groups.

Table 2 presents the results of further subgroup analysis to investigate the baseline differences among the M-TURP, B-TURP, PVP, ThuVARP, HoLEP, and DiLEP groups (Table 2). No differences in common comorbidities and medication history were observed among the six groups.

### 3.2. Comparison of the Length of Hospital Stay among Different Surgery Types

**Figure 2:**
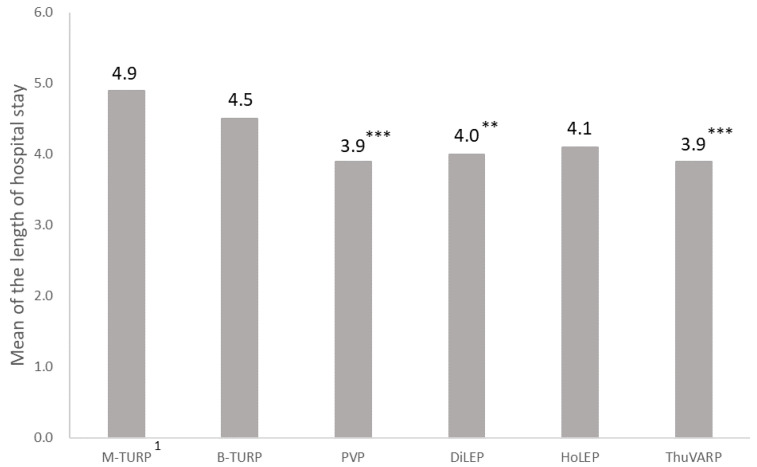
Average length of hospital stay following TURP and laser surgery. ** *p* < 0.01, and *** *p* < 0.001 versus M-TURP. ^1^ M-TURP was the reference group.

Patients were stratified into six subgroups, and the groups were balanced using PSM. Significantly fewer inpatient days were observed in the PVP (3.9 ± 2.5 days, *p* < 0.001), ThuVARP (3.9 ± 2.7 days, *p* < 0.001), HoLEP (4.1 ± 1.3 days, *p* = 0.663), DiLEP (4.0 ± 1.9 days, *p* = 0.002), and B-TURP (4.5 ± 3.8 days, *p* = 0.283) groups than in the M-TURP (4.9 ± 4.3 days) group (Figure 2).

### 3.3. Comparison of ER Visits with Rehospitalization Due to Postoperative Bleeding

We estimated the proportion of patients who returned to our ER due to clot retention within 15, 30, 60, and 90 days after surgery (Table 3).

The percentage of patients who returned to our ER due to bleeding events within 90 days after surgery was the highest in the HoLEP group (17.4%, *p* = 0.104), followed by the DiLEP (15.2%, *p* = 0.026), B-TURP (10.5%, *p* = 0.934), PVP (9.6%, *p* = 0.387), ThuVARP (9.4%, *p* = 0.257), and M-TURP (10.4%, reference) groups.

The percentage of patients who were rehospitalized for bleeding events within 90 days after surgery was the highest in the DiLEP group (8.9%, *p* = 0.714), followed by the M-TURP (8.1%, reference), B-TURP (6.0%, *p* = 0.119), ThuVARP (5.5%, *p* = 0.016), PVP (5.2%, *p* = 0.039), and HoLEP (N/A, *p* = 0.277) groups.

### 3.4. Effect of Surgery Type on Bleeding Events

The effect of six surgeries on the risk of bleeding events was assessed using the multivariate Cox regression analysis with adjustment for age, sex, CCI, comedications (statins, ACEIs, β-blockers, anticoagulants, antiplatelets, spironolactone, and 5-α-reductase inhibitors), comorbidities (HCD, hyperlipidemia, DM, and CKD), and the index year at the start of follow-up.

We evaluated the bleeding events leading to ER visits within 90 days after surgery. The adjusted HRs of the PVP (0.92; 95% CI, 0.63–1.34, *p* = 0.677) and ThuVARP (0.91; 95% CI, 0.68–1.20, *p* = 0.493) groups were lower than that of the M-TURP group; however, the difference was nonsignificant. The HRs of DiLEP (1.52; 95% CI, 1.06–2.16, *p* = 0.022), HoLEP (1.70; 95% CI, 0.83–3.50, *p* = 0.150), and B-TURP (1.04; 95% CI, 0.76–1.41, *p* = 0.826) groups were higher than that of the M-TURP group (Table 4).

The analysis of the bleeding visits leading to rehospitalization within 90 days after surgery revealed that the adjusted HRs of the PVP (0.61; 95% CI, 0.38–1.00 *p* = 0.050) and ThuVARP (0.67; 95% CI, 0.47–0.95, *p* = 0.024) groups were significantly lower than that of the M-TURP group. Moreover, the HR of the DiLEP (1.12; 95% CI, 0.71–1.75, *p* = 0.634) group was higher than that of the M-TURP group (Table 5).

**Figure 3:**
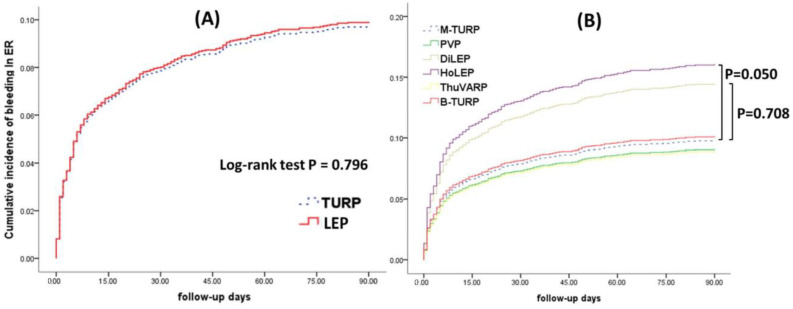
(**A**) Cumulative incidence of emergency room (ER) visits with postoperative bleeding post TURP and laser surgery. (**B**) Cumulative incidence of ER visits due to postoperative bleeding post different surgery groups. Abbreviations: M-TURP, Monopolar transurethral resection of the prostate; B-TURP, Bipolar transurethral resection of the prostate; PVP, GreenLight Photo vaporization of the prostate; ThuVARP, Thulium Laser Vaporesection of the Prostate; HoLEP, Holmium laser enucleation of the prostate; DiLEP, Diode laser (980 nm) enucleation of the Prostate.

Regarding bleeding events leading to ER visits, the 90-day cumulative incidence following laser surgery was not significantly different from that of TURP (*p* = 0.796; Figure 3A), and the same was verified between PVP, ThuVARP, and B-TURP and M-TURP. Moreover, the bleeding risk following HoLEP and DiLEP was higher than after M-TURP (*p* = 0.050, *p* = 0.708; Figure 3B).

**Figure 4:**
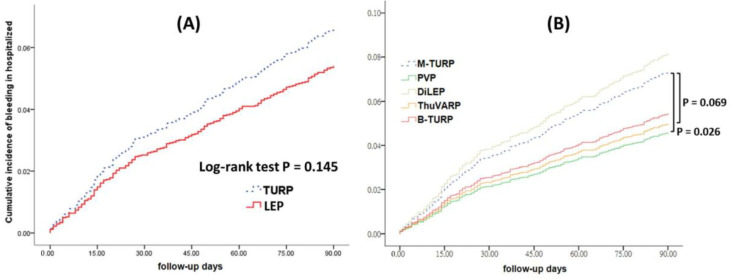
(**A**). Cumulative incidence of rehospitalization due to postoperative bleeding after TURP and laser surgery. (**B**). Cumulative incidence of rehospitalization due to postoperative bleeding in different surgery groups. Abbreviations: M-TURP, Monopolar transurethral resection of the prostate; B-TURP, Bipolar transurethral resection of the prostate; PVP, GreenLight Photo vaporization of the prostate; ThuVARP, Thulium Laser Vaporesection of the Prostate; HoLEP, Holmium laser enucleation of the prostate; DiLEP, Diode laser (980 nm) enucleation of the Prostate.

Regarding the bleeding events leading to rehospitalization, the 90-day cumulative incidence post-laser surgery was not significantly different after TURP (*p* = 0.145; Figure 4A). The cumulative incidence of bleeding events leading to rehospitalization was lower in the PVP and ThuVARP groups than that of the M-TURP cohort (*p* = 0.026, *p* = 0.069; Figure 4B).

### 3.5. Comparison of Postoperative Bleeding between the Subgroups of Demographics, Comorbidities, and Comedications

Significantly higher rates of clot retention leading to rehospitalization were noted in patients aged >80 years (2.93; 95% CI, 1.52–5.65, *p* = 0.001). A high CCI score (>4) was observed in patients visiting the ER due to bleeding events after 90 days of surgery (2.36; 95% CI, 1.43–3.92, *p* = 0.001). No significant differences in bleeding events were observed between patients visiting the ER and those who were rehospitalized in the subgroups of comorbidities (DM, HCD, CKD, hyperlipidemia, and heart failure) and comedications (statins, ACEIs, β-blockers, anticoagulants, antiplatelets, spironolactone, and 5-α-reductase inhibitors).

## 4. Discussion

To the best of our knowledge, our study is the first to compare six transurethral procedures for the treatment of BPH or lower urinary tract infection and to investigate differences in postoperative bleeding. No study has compared six transurethral procedures by using balanced baseline characteristics.

### 4.1. Participants in Balanced Groups

We presented the results of laser surgery and TURP with adjustments for demographics, comorbidities, and comedications by using PSM. Most studies did not perform a multivariate analysis with adjustments for confounders [5]. We adjusted the outcomes between the age, comorbidities, and comedication cohorts. In order to avoid the inconsistency of comorbidities, we used CCI [23].

### 4.2. Main Results

Most studies reported shorter hospitalization durations with PVP, ThuVARP, DiLEP, and HoLEP than with TURP [24,25,26,27]. Some smaller studies reported no significant differences in hospitalization duration between HoLEP and B-TURP [25,28]. Our study revealed significantly shorter hospitalization durations with PVP, ThuVARP, and DiLEP than with M-TURP but not with HoLEP and B-TURP.

Shamout et al. reported that 28.6% and 11.1% of the patients returned to ER or were rehospitalized post-M-TURP, respectively. This was higher than in our study [29]. In another study, the reported the percentage of patients who returned to ER or were rehospitalized post-M-TURP (8.0%, 2.8%), HoLEP (9.8%, 0.9%), and PVP (7.5%, 1.7%) was lower than in our study [30].

The evidence involving ThuVARP and DiLEP and their bleeding risk and other complications is still scarce [31]. Therefore, we believe that a complete follow-up study of all six transurethral procedures is needed to be able to fully compare and balance their risk/benefit.

### 4.3. Differences in Postoperative Bleeding between Different Surgery Types

Early postoperative bleeding is a frequent complication occurring within 1 to 3 months after BPH surgery [32,33] and is treated with bladder irrigation and clot removal, if necessary [34].

PVP and ThuVARP are safer than M-TURP because blood transfusion, clot retention, hemoglobin decline, or transurethral resection syndrome is less likely to occur [35]. Our findings support changes in the surgical treatment of BPH from M-TURP to new laser methods [24,36,37]. However, DiLEP and HoLEP resulted in more bleeding events than M-TURP. Early DiLEP and HoLEP therapies are associated with increased postoperative complications, although some later studies reported lower morbidity in patients undergoing these therapies [38,39].

### 4.4. Comparison between the Subgroups of Demographics, Comorbidities, and Comedications

The high CCI score subgroup demonstrated increased HRs for ER visits within 90 days after surgery, whereas patients aged >80 years exhibited a significant risk of bleeding leading to rehospitalization. Our study suggested that laser surgery is safer than M-TURP for BPH because of reduced catheter time and risk of bleeding, even in patients receiving anticoagulant or antiplatelet therapy [23,35,40].

We investigated four types of laser surgery and two types of TURP and demonstrated that laser therapies are associated with a shorter hospital stay, less bleeding, and lower transfusion requirements than TURP, even in patients receiving anticoagulant or antiplatelet therapy [41,42]. However, the rates of ER visits and rehospitalization revealed that DiLEP and HoLEP were not associated with reduced bleeding complications [43,44]. In the recent decade, favorable outcomes were obtained after laser surgery, but a high percentage of urologists still prefer TURP. This may be because of the perceived precipitous learning curve of laser surgery [45,46,47].

### 4.5. Limitations

First, we used a retrospective cohort design. We matched all potential confounders between the surgery cohorts, but the selection and observational bias may still exist. Meanwhile, the sample size differs between all sub-groups studied, and this may result in bias. However, the use of data from the NHIRD, a large and well-validated database, may control the bias.

The second limitation is the accuracy of diagnosis. Potential misdiagnosis exists in the NHIRD because of the possible misclassification of *ICD-9-CM* and *ICD-10-CM* codes.

Third, the NHIRD lacks specific clinical (e.g., the weight of the resected prostate, catheterization time, and hemoglobin decline) and lifestyle behavior (e.g., Body Mass Index, smoking behavior, and alcohol use) data.

Finally, the study lacks information regarding the surgeons’ experience and training.

These limitations do not compromise the conclusions of this study. However, large-scale prospective studies should be conducted to further validate our results.

## 5. Conclusions

Among the six investigated, transurethral procedures for BPH, PVP, and ThuVARP were safer than M-TURP because bleeding events and clot retention were less likely to occur, even in patients receiving anticoagulant or antiplatelet therapy. However, DiLEP and HoLEP did not result in fewer bleeding events than M-TURP. Our findings suggest that PVP and ThuVARP are effective alternatives to M-TURP for the surgical treatment of BPH. These results would provide useful information for urologists and patients with BPH.

## Figures and Tables

**Table 1:** Baseline characteristics of patients who underwent laser surgery or TURP.

		Laser	%	TURP	%	*p*
Age, year (mean ± SD)		70.9 ± 8.4		70.9 ± 8.4		1.000
Age	50–59	145	8.8	145	8.8	1.000
	60–69	605	36.6	605	36.6	
	70–79	594	36.0	594	36.0	
	>80	307	18.6	307	18.6	
CCI (mean ± SD)		3.2 ± 2.6		3.2 ± 2.6		1.000
CCI	0	258	15.6	258	15.6	1.000
	1–2	513	31.1	513	31.1	
	3–4	397	24.0	397	24.0	
	>4	483	29.3	483	29.3	
DM	N	1162	70.4	1162	70.4	1.000
	Y	489	29.6	489	29.6	
HCD	N	542	32.8	542	32.8	1.000
	Y	1109	67.2	1109	67.2	
CKD	N	1471	89.1	1471	89.1	1.000
	Y	180	10.9	180	10.9	
Hyperlipidemia	N	793	48.0	793	48.0	1.000
	Y	858	52.0	858	52.0	
Heart failure	N	1066	64.6	1086	65.8	0.465
	Y	585	35.4	565	34.2	
Statins	N	1008	61.1	1029	62.3	0.452
	Y	643	38.9	622	37.7	
ACEI	N	856	51.8	856	51.8	1.000
	Y	795	48.2	795	48.2	
β-blocker	N	628	38.0	645	39.1	0.543
	Y	1023	62.0	1006	60.9	
Anticoagulant	N	1527	92.5	1506	91.2	0.182
	Y	124	7.5	145	8.8	
Antiplatelet	N	736	44.6	755	45.7	0.506
	Y	915	55.4	896	54.3	
Spironolactone	N	1491	90.3	1476	89.4	0.387
	Y	160	9.7	175	10.6	
5α reductase inhibitors	N	992	60.1	1043	63.2	0.068
	Y	659	39.9	608	36.8	

Abbreviations: CCI, Charlson comorbidity index; HCD, hypertensive cardiovascular disease; DM, diabetes mellitus; CKD, chronic kidney disease; ACEI, angiotensin-converting enzyme inhibitor.

**Table 2:** Baseline characteristics of patients who underwent different LEPs or TURPs.

		M-TURP	%	PVP	%	DiLEP	%	HoLEP	%	ThuVARP	%	B-TURP	%	*p*
Age	50–59	100	9.9	31	8.1	28	9.9	5	10.9	81	8.6	45	7.1	0.854
	60–69	355	35	136	35.4	112	39.7	16	34.8	341	36.3	250	39.2	
	70–79	367	36.2	138	35.9	94	33.3	17	37	345	36.7	227	35.6	
	>80	192	18.9	79	20.6	48	17	8	17.4	172	18.3	115	18.1	0.898
CCI	0	147	14.5	54	14.1	42	14.9	7	15.2	155	16.5	111	17.4	
	1–2	325	32.1	119	31	80	28.4	12	26.1	302	32.2	188	29.5	
	3–4	239	23.6	92	24	76	27	13	28.3	216	23	158	24.8	
	>4	303	29.9	119	31	84	29.8	14	30.4	266	28.3	180	28.3	
DM	N	705	69.5	269	70.1	197	69.9	28	60.9	668	71.1	457	71.7	0.664
	Y	309	30.5	115	29.9	85	30.1	18	39.1	271	28.9	180	28.3	
HCD	N	330	32.5	122	31.8	98	34.8	13	28.3	309	32.9	212	33.3	0.945
	Y	684	67.5	262	68.2	184	65.2	33	71.7	630	67.1	425	66.7	
CKD	N	900	88.8	341	88.8	253	89.7	37	80.4	840	89.5	571	89.6	0.53
	Y	114	11.2	43	11.2	29	10.3	9	19.6	99	10.5	66	10.4	
Hyperlipidemia	N	485	47.8	189	49.2	133	47.2	15	32.6	456	48.6	308	48.4	0.436
	Y	529	52.2	195	50.8	149	52.8	31	67.4	483	51.4	329	51.6	
Heart failure	N	674	66.5	246	64.1	182	64.5	30	65.2	608	64.7	412	64.7	0.949
	Y	340	33.5	138	35.9	100	35.5	16	34.8	331	35.3	225	35.3	
Statins	N	624	61.5	232	60.4	181	64.2	23	50	572	60.9	405	63.6	0.431
	Y	390	38.5	152	39.6	101	35.8	23	50	367	39.1	232	36.4	
ACEI	N	536	52.9	179	46.6	150	53.2	21	45.7	506	53.9	320	50.2	0.17
	Y	478	47.1	205	53.4	132	46.8	25	54.3	433	46.1	317	49.8	
β-blocker	N	378	37.3	156	40.6	91	32.3	18	39.1	363	38.7	267	41.9	0.104
	Y	636	62.7	228	59.4	191	67.7	28	60.9	576	61.3	370	58.1	
Anticoagulant	N	923	91	340	88.5	263	93.3	N/A ^1^	N/A	880	93.7	583	91.5	0.027
	Y	91	9	44	11.5	19	6.7	N/A	N/A	59	6.3	54	8.5	
Antiplatelet	N	448	44.2	161	41.9	122	43.3	18	39.1	435	46.3	307	48.2	0.308
	Y	566	55.8	223	58.1	160	56.7	28	60.9	504	53.7	330	51.8	
Spironolactone	N	902	89	348	90.6	256	90.8	43	93.5	844	89.9	574	90.1	0.832
	Y	112	11	36	9.4	26	9.2	3	6.5	95	10.1	63	9.9	
5α reductase	N	655	64.6	244	63.5	162	57.4	27	58.7	559	59.5	388	60.9	0.13
inhibitor	Y	359	35.4	140	36.5	120	42.6	19	41.3	380	40.5	249	39.1	

^1^ N/A: Number of bleeding events in the HoLEP group was <3. According to the data protection policy of NHIRD, data less than 3 cannot be provided. Abbreviations: CCI, Charlson comorbidity index; HCD, hypertensive cardiovascular disease; DM, diabetes mellitus; CKD, chronic kidney disease; ACEI, angiotensin-converting enzyme inhibitor.

**Table 3:** Postoperative bleeding events in patients who underwent different LEPs or TURPs.

	M-TURP ^1^ (*n* = 1014)	%	PVP (*n* = 384)	%	*p*	DiLEP (*n* = 282)	%	*p*	HoLEP ^2^ (*n* = 46)	%	*p*	ThuVARP (*n* = 939)	%	*p*	B-TURP (*n* = 637)	%	*p*
Emergency room ^3^																	
15 days	68	6.7	28	7.3	0.389	33	11.7	**0.008**	5	10.9	0.239	60	6.4	0.425	44	6.9	0.92
30 days	86	8.5	30	7.8	0.388	37	13.1	**0.022**	6	13	0.28	72	7.7	0.283	51	8	0.784
60 days	98	9.7	35	9.1	0.421	43	15.2	**0.009**	8	17.4	0.124	83	8.8	0.291	65	10.2	0.735
90 days	105	10.4	37	9.6	0.387	43	15.2	**0.026**	8	17.4	0.104	88	9.4	0.257	67	10.5	0.934
Rehospitalization ^4^																	
15 days	23	2.3	7	1.8	0.391	5	1.8	0.817	N/A	N/A	0.72	15	1.6	0.182	10	1.6	0.37
30 days	41	4	13	3.4	0.346	9	3.2	0.602	N/A	N/A	0.446	25	2.7	0.059	14	2.2	0.048
60 days	59	5.8	17	4.4	0.187	19	6.7	0.572	N/A	N/A	0.251	40	4.3	0.071	29	4.6	0.311
90 days	82	8.1	20	5.2	**0.039**	25	8.9	0.714	N/A	N/A	0.277	52	5.5	**0.016**	38	6	0.119

^1^ M-TURP was the reference method. ^2^ N/A: Number of bleeding events in the HoLEP group was <3. According to the data protection policy of NHIRD, data less than 3 cannot be provided. ^3^ Bleeding events leading to emergency room visits. ^4^ Bleeding events leading to rehospitalization. *p* values marked in bold indicate statistically significant differences between the groups. Abbreviations: CCI, Charlson comorbidity index; HCD, hypertensive cardiovascular disease; DM, diabetes mellitus; CKD, chronic kidney disease; ACEI, angiotensin-converting enzyme inhibitor; M-TURP, Monopolar transurethral resection of the prostate; B-TURP, Bipolar transurethral resection of the prostate; PVP, GreenLight Photo vaporization of the prostate; ThuVARP, Thulium Laser Vaporesection of the Prostate; HoLEP, Holmium laser enucleation of the prostate; DiLEP, Diode laser (980 nm) enucleation of the Prostate. ACEI, angiotensin-converting enzyme inhibitor; HR, hazard ratio.

**Table 4:** Adjusted hazard ratios for emergency room visits due to postoperative bleeding after 15, 30, 60, and 90 days of surgery.

	15 Days		30 Days		60 Days		90 Days	
	HR (95% CI)	*p*	HR (95% CI)	*p*	HR (95% CI)	*p*	HR (95% CI)	*p*
Age								
50–59 (As Reference)								
60–69	0.94 (0.55–1.60)	0.824	0.98 (0.60–1.60)	0.941	1.00 (0.63–1.59)	0.992	1.03 (0.66–1.62)	0.894
70–79	0.98 (0.58–1.68)	0.947	0.95 (0.58–1.55)	0.832	1.14 (0.72–1.81)	0.585	1.12 (0.71–1.77)	0.615
>80	1.11 (0.63–1.97)	0.715	1.12 (0.66–1.88)	0.684	1.29 (0.79–2.11)	0.316	1.32 (0.81–2.13)	0.265
Charlson Comorbidity Index								
0 (As Reference)								
1–2	1.87 (1.06–3.29)	**0.03**	1.91 (1.14–3.20)	**0.014**	1.92 (1.20–3.08)	**0.007**	1.97 (1.24–3.12)	**0.004**
3–4	2.01 (1.10–3.67)	**0.023**	1.88 (1.08–3.28)	**0.026**	1.96 (1.19–3.25)	**0.009**	2.06 (1.26–3.37)	**0.004**
>4	2.35 (1.27–4.34)	**0.006**	2.37 (1.35–4.16)	**0.003**	2.30 (1.37–3.85)	**0.002**	2.36 (1.43–3.92)	**0.001**
DM	0.86 (0.63–1.17)	0.348	0.98 (0.74–1.30)	0.89	1.03 (0.80–1.33)	0.821	1.07 (0.83–1.37)	0.605
HCD	1.23 (0.83–1.83)	0.301	1.25 (0.88–1.80)	0.217	1.19 (0.86–1.66)	0.292	1.13 (0.82–1.56)	0.442
CKD	1.21 (0.84–1.76)	0.308	1.25 (0.89–1.75)	0.203	1.15 (0.84–1.58)	0.392	1.15 (0.84–1.57)	0.39
Hyperlipidemia	1.14 (0.82–1.58)	0.44	1.04 (0.77–1.41)	0.779	1.00 (0.75–1.32)	0.999	1.01 (0.76–1.32)	0.967
Heart failure	1.06 (0.78-1.44)	0.702	1.01 (0.76-1.34)	0.947	1.01 (0.78-1.31)	0.95	1.03 (0.80-1.33)	0.803
Statins	0.96 (0.70-1.32)	0.802	1.02 (0.76-1.36)	0.911	1.08 (0.82-1.41)	0.598	1.03 (0.79-1.34)	0.835
ACEI	1.08 (0.79–1.48)	0.615	1.03 (0.77–1.36)	0.852	1.03 (0.79–1.34)	0.82	1.08 (0.83–1.40)	0.569
β-blocker	1.38 (1.00–1.92)	0.05	1.39 (1.03–1.87)	**0.033**	1.29 (0.98–1.69)	0.065	1.26 (0.96–1.64)	0.09
Anticoagulant	0.64 (0.39–1.08)	0.093	0.75 (0.48–1.17)	0.201	0.69 (0.45–1.05)	0.084	0.69 (0.45–1.04)	0.077
Antiplatelet	1.59 (0.76–3.34)	0.217	1.34 (0.68–2.67)	0.4	1.43 (0.76–2.68)	0.268	1.28 (0.69–2.37)	0.44
Spironolactone	1.20 (0.82–1.76)	0.353	1.22 (0.86–1.73)	0.262	1.28 (0.93–1.76)	0.136	1.23 (0.90–1.69)	0.195
5α reductase inhibitors	1.20 (0.93–1.55)	0.165	1.20 (0.95–1.52)	0.129	1.17 (0.94–1.45)	0.162	1.13 (0.91–1.40)	0.254
Surgery type								
M-TURP (As Reference)								
PVP	1.09 (0.70–1.69)	0.712	0.92 (0.61–1.40)	0.708	0.94 (0.64–1.38)	0.745	0.92 (0.63–1.34)	0.677
DiLEP	1.74 (1.15–2.65)	**0.009**	1.57 (1.06–2.31)	**0.023**	1.63 (1.13–2.33)	**0.008**	1.52 (1.06–2.16)	**0.022**
HoLEP	1.59 (0.64–3.96)	0.319	1.53 (0.67–3.51)	0.315	1.82 (0.88–3.74)	0.106	1.70 (0.83–3.50)	0.15
ThuVARP	0.95 (0.67–1.35)	0.774	0.91 (0.66–1.24)	0.544	0.91 (0.68–1.22)	0.545	0.91 (0.68–1.20)	0.493
B-TURP	1.04 (0.71–1.53)	0.829	0.97 (0.68–1.37)	0.842	1.08 (0.79–1.48)	0.645	1.04 (0.76–1.41)	0.826

*p*-values marked in bold indicate statistically significant differences between the groups. Abbreviations: CCI, Charlson comorbidity index; DM, diabetes mellitus; HCD, hypertensive cardiovascular disease; CKD, chronic kidney disease; ACEI, angiotensin-converting enzyme inhibitor; M-TURP, Monopolar transurethral resection of the prostate; B-TURP, Bipolar transurethral resection of the prostate; PVP, GreenLight Photo vaporization of the prostate; ThuVARP, Thulium Laser Vaporesection of the Prostate; HoLEP, Holmium laser enucleation of the prostate; DiLEP, Diode laser (980nm) enucleation of the Prostate. ACEI, angiotensin-converting enzyme inhibitor; HR, hazard ratio.

**Table 5:** Adjusted hazard ratios for rehospitalization due to postoperative bleeding after 15, 30, 60, and 90 days of surgery.

	15 Days		30 Days		60 Days		90 Days	
	HR (95% CI)	*p*	HR (95% CI)	*p*	HR (95% CI)	*p*	HR (95% CI)	*p*
Age								
50–59 (As Reference)								
60–69	1.61 (0.37–7.11)	0.528	1.39 (0.54–3.60)	0.498	1.38 (0.65–2.93)	0.408	1.21 (0.63–2.31)	0.571
70–79	2.63 (0.61–11.2)	0.194	1.43 (0.55–3.73)	0.46	1.58 (0.74–3.35)	0.238	1.68 (0.88–3.19)	0.115
>80	2.69 (0.6–12.14)	0.198	2.20 (0.83–5.87)	0.114	2.69 (1.24–5.81)	**0.012**	2.93 (1.52–5.65)	**0.001**
Charlson Comorbidity Index								
0 (As Reference)								
1–2	1.19 (0.41–3.44)	0.752	1.00 (0.47–2.11)	0.998	1.01 (0.58–1.77)	0.973	1.06 (0.64–1.73)	0.829
3–4	1.34 (0.43–4.18)	0.609	1.21 (0.54–2.70)	0.638	1.07 (0.58–1.97)	0.84	1.14 (0.66–1.95)	0.641
>4	1.82 (0.57–5.77)	0.309	1.49 (0.65–3.40)	0.349	1.25 (0.66–2.38)	0.496	1.15 (0.65–2.03)	0.639
DM	1.15 (0.63–2.10)	0.654	1.08 (0.67–1.73)	0.748	1.08 (0.74–1.58)	0.688	1.20 (0.87–1.66)	0.274
HCD	0.67 (0.32–1.42)	0.296	0.97 (0.54–1.73)	0.917	0.81 (0.52–1.27)	0.362	0.77 (0.52–1.14)	0.188
CKD	0.98 (0.46–2.10)	0.962	1.39 (0.80–2.39)	0.24	1.39 (0.89–2.16)	0.149	1.44 (0.98–2.11)	0.065
Hyperlipidemia	1.00 (0.53–1.88)	0.988	0.87 (0.53–1.43)	0.585	0.80 (0.54–1.20)	0.28	0.92 (0.65–1.30)	0.633
Heart failure	1.48 (0.79–2.79)	0.219	1.14 (0.71–1.83)	0.598	1.24 (0.85–1.82)	0.265	1.35 (0.97–1.89)	0.079
Statins	0.69 (0.36–1.31)	0.255	0.79 (0.48–1.30)	0.35	0.92 (0.61–1.37)	0.667	0.88 (0.63–1.25)	0.478
ACEI	0.89 (0.48–1.65)	0.706	0.90 (0.56–1.46)	0.68	0.99 (0.68–1.44)	0.952	1.04 (0.75–1.45)	0.795
β-blocker	1.19 (0.62–2.28)	0.602	1.18 (0.72–1.92)	0.518	0.99 (0.68–1.44)	0.956	1.11 (0.80–1.55)	0.529
Anticoagulant	1.33 (0.63–2.82)	0.454	1.21 (0.65–2.24)	0.547	1.01 (0.59–1.71)	0.977	0.91 (0.56–1.46)	0.685
Antiplatelet	1.58 (0.35–7.21)	0.557	0.88 (0.30–2.64)	0.823	1.25 (0.53–2.96)	0.614	1.29 (0.61–2.73)	0.501
Spironolactone	1.09 (0.52–2.29)	0.816	1.34 (0.77–2.32)	0.295	1.14 (0.72–1.81)	0.582	1.16 (0.78–1.72)	0.475
5α reductase inhibitors	1.65 (1.00–2.74)	0.052	1.48 (1.01–2.19)	**0.047**	1.56 (1.14–2.11)	**0.005**	1.30 (0.99–1.69)	0.057
Surgery type								
M-TURP (As Reference)								
PVP	0.77 (0.33–1.80)	0.55	0.82 (0.44–1.53)	0.526	0.72 (0.42–1.24)	0.243	0.61 (0.38–1.00)	**0.05**
DiLEP	0.75 (0.28–1.98)	0.561	0.78 (0.38–1.61)	0.5	1.15 (0.69–1.94)	0.59	1.12 (0.71–1.75)	0.634
HoLEP	1.00 (0.13–7.45)	0.996	0.57 (0.08–4.19)	0.584	0.38 (0.05–2.74)	0.337	0.54 (0.13–2.22)	0.397
ThuVARP	0.69 (0.36–1.33)	0.271	0.65 (0.39–1.07)	0.089	0.71 (0.47–1.06)	0.092	0.67 (0.47–0.95)	**0.024**
B-TURP	0.70 (0.33–1.47)	0.346	0.54 (0.29–1.00)	**0.049**	0.77 (0.49–1.20)	0.249	0.73 (0.50–1.08)	0.117

*p*-values marked in bold indicate statistically significant differences between the groups. Abbreviations: CCI, Charlson comorbidity index; DM, diabetes mellitus; HCD, hypertensive cardiovascular disease; CKD, chronic kidney disease; ACEI, angiotensin-converting enzyme inhibitor; M-TURP, Monopolar transurethral resection of the prostate; B-TURP, Bipolar transurethral resection of the prostate; PVP, GreenLight Photo vaporization of the prostate; ThuVARP, Thulium Laser Vaporesection of the Prostate; HoLEP, Holmium laser enucleation of the prostate; DiLEP, Diode laser (980 nm) enucleation of the Prostate. ACEI, angiotensin-converting enzyme inhibitor; HR, hazard ratio.

## Data Availability

The personal electronic data of NHIRD protects by the Computer-Processed Personal Data Protection Law. The results of the academic study are available for researchers from the NHIRD of Taiwan. All researchers accord with the criteria for access to confidential data, which cannot be shared publicly because of legal guidelines imposed by the government of Taiwan under the “Personal Information Protection Act”. Requests for data can be applied as an official proposal to the NHIRD (https://dep.mohw.gov.tw/dos/np-2497-113.html (accessed on 7 August 2022)). The contact information for needed data is: 886-2-85906828; Email: sthuiying@mohw.gov.tw.

## Abbreviations

**Abbreviation**	**Meaning**
ACEIs	angiotensin-converting enzyme inhibitors
BPH	benign prostatic hyperplasia
B-TURP	bipolar transurethral resection of the prostate
CCI	Charlson comorbidity index
CI	confidence interval
CKD	chronic kidney disease
DiLEP	diode laser (980 nm) enucleation of the prostate
DM	diabetes mellitus
ER	emergency room
HCD	hypertensive cardiovascular disease
HoLEP	holmium laser enucleation of the prostate
HR	hazard ratio
ICD-10-CM	International Classification of Diseases, Ten Revision, Clinical Modification
ICD-9-CM	International Classification of Diseases, Ninth Revision, Clinical Modification
IRB	Institutional Review Board
M-TURP	monopolar transurethral resection of the prostate
NHI	National Health Insurance
NHIRD	National Health Insurance Research Database
PSM	propensity score matching
PVP	GreenLight photovaporization of the prostate
SD	standard deviation
ThuVARP	thulium laser vaporesection of the prostate
TURP	transurethral resection of the prostate
